# Editorial: What is new on the horizon in neonatology? Recent advances in monitoring, diagnostics, and therapeutics in neonatal care

**DOI:** 10.3389/fped.2025.1552262

**Published:** 2025-01-13

**Authors:** Minesh Khashu, Karel Allegaert

**Affiliations:** ^1^ Department of Neonatology, University Hospitals Dorset NHS Foundation Trust, Dorset, United Kingdom; ^2^ Department of Development and Regeneration, KU Leuven, Leuven, Belgium; ^3^ Department of Pharmaceutical and Pharmacological Sciences, KU Leuven, Leuven, Belgium; ^4^ Department of Hospital Pharmacy, Erasmus MC, Rotterdam, Netherlands

**Keywords:** medical device, NICU, pharmacotherapy, equity, diagnostics, therapeutics

**Editorial on the Research Topic**
What is new on the horizon in neonatology? Recent advances in monitoring, diagnostics, and therapeutics in neonatal care

## Introduction

Neonates and infants are commonly referred to as “therapeutic orphans” due to the overall scarcity of therapeutic interventions that have been developed and tailored to their needs and specific characteristics (1, 2). This is well known by care providers and researchers active in this field, but is perhaps less on the radar of authorities, funding bodies or the broader public. There is significant health inequity when comparing newborns to other age populations in terms of specific drug and device development and therapeutics (1, 2). In addition there are health inequities in the provision of neonatal care globally which require special attention in terms of improvement (3).

Bronchopulmonary dysplasia, neonatal seizures, poor growth, necrotizing enterocolitis (NEC) and short bowel, hypoxic-ischemic encephalopathy, retinopathy of prematurity (ROP), neonatal infections and sepsis hereby serve as a non-exhaustive list of “orphan conditions” in need of more equity, through adequately and urgently funded research and improvement.

The good news is that there have been increased efforts, in recent years, by researchers and regulatory bodies to focus on the provision of drugs, devices, and treatment modalities tailored for neonatal use, while further advocacy remains an obvious need (2, 4, 5). This brings perspective and explains the initiative taken to organize a focused research topic on what is on the horizon as well as recent advances.

## Overview of the topics covered

We targeted emerging or new aspects related to monitoring, diagnostics and therapeutics in neonatal care for the current research topic. Fortunately, this research topic was perceived as very relevant by the research community, as 135 authors expressed their interest as contributors, resulting in 20 accepted papers. This serves as a signal of the importance to continue to work on this topic.

Post-hoc, and in a somewhat arbitrary way (because of overlap in these subcategories) these papers were subdivided by the editors into different subcategories, with focus on (*1*, 5 papers) perinatal biomarkers in blood and urine and how these relate to or predict outcomes, (*2*, 6 papers) adaptations of existing and newly emerging equipment in neonatal units, (*3*, 3 papers) needed advances in pharmacotherapy, (*4*, 3 papers) machine learning or deep learning applications in neonatal care, and finally, (*5*, 3 papers) underreported aspects of contemporary NICU care, with a focus on the holistic nature of care for the infant and the family.

### Perinatal biomarkers in blood and urine and how these relate to or predict outcomes

Two papers focused on biomarkers related to gestational diabetes, with reflections and data on maternal and neonatal outcomes. Postnatal maternal levels of glycated albumin and hemoglobin A1c in mothers of large-for-gestational-age (LGA) informed us of the relevance of accurate diagnosis during pregnancy. This is because postpartum women without diagnosis during pregnancy had higher glycated albumin values, associated with LGA and associated complications (Železnik et al.). Interestingly and related to this paper, Yin et al. reported on a untargeted metabolomics study in women with gestational diabetes, with the recommendation of a maternal serum metabolite panel to forecast neonatal adverse outcomes (hypoglycemia and macrosomia) (Yin et al.).

Other papers focused on the use of vitamin D, acid-base and biomarkers associated with fetal growth restriction with impaired neurodevelopmental outcome. In a cohort of 217 preterm neonates, a multivariate regression analysis identified antenatal steroids as protective, and lower birth weight, duration of ventilation, sepsis and the serum 25-(OH)D vitamin as risk factors to develop ROP (Yin et al.). Musco et al. reported on a systematic review on blood biomarkers indicating risks of adverse neurodevelopmental outcome in fetal growth restricted infants (Musco et al.). While the authors retrieved some data on neuron specific enolase and S100B, the overall conclusions reflect a call for further research. Finally, an association between lactate levels in umbilical cord blood and cerebral oxygenation in preterm neonates was studied as a secondary outcome analysis (Dusleag et al.). In non-asphyxiated preterm neonates with respiratory support, lactate levels were negatively associated with cerebral and arterial oxygenation. In term neonates without respiratory support, no associations were observed.

### Adaptations of existing and newly emerging equipment in our units

In a review on emerging innovations in neonatal monitoring, Krbec et al. concluded that there is an urgent, still unmet need to develop wireless, non- or minimal-contact, non-adhesive technology, capable to integrate multiple signals in a single platform, tailored to neonates (Krbec et al.). Related to this call of action, Svoboda et al. reported on their pilot experience with contactless assessment of heart rate, applying imaging photoplethysmography (Svoboda et al.). Rectal and axillary temperature monitoring on admission were compared in a cohort of preterm (*n* = 80, <32 weeks gestational age) by Halabi et al., reporting that rectal measurement was likely more reliable in the event of hypothermia (Halabi et al.). Ultrasound-guided measurement of anterior cerebral artery resistive index in the first week of life in 739 preterm neonates (<35 weeks) was not associated with subsequent co-morbidities on admission or during neonatal stay (asphyxia, sepsis, NEC) (Singh Gill et al.). A case series of neurally adjusted ventilatory assist to rescue pulmonary interstitial emphysema in 5 extremely low birth weight infants illustrated the potential value of this ventilatory equipment and strategy and need for further study (Chen et al.). Finally, van Rens et al. compared a conventional to a modified Seldinger technique (a dedicated micro-insertion kit) for peripherally inserted central catheter (PICC) placement, illustrating the relevance of developing “low risk, high benefit” type of medical devices, adapted to the specific needs of neonates (van Rens et al.).

### Advances needed in pharmacotherapy

The currently available medicines and dosing regimens in neonatal care are limited and there is an urgent need for improvement in this domain. This was illustrated by articles on sepsis, septic shock and steroids. Inequity in provision of neonatal care across the globe ought to be a major focus of improvement. Gezahegn et al. described the outcome in neonates admitted with sepsis in Harar (Ethiopia). Low white blood cell count, desaturation, preterm birth, absence of prenatal maternal care, and chorioamnionitis were important risk factors for sepsis-related mortality (Gezahegn et al.). Addressing these prognostic factors hold the promise to act as levelers to improve outcomes. A pilot study compared noradrenaline and adrenaline as first line vasopressor for fluid-refractory sepsis shock (Garegrat et al.). Both interventions were comparable to resolve the septic shock, while the overall mortality (13/42, 30%) remained significant, highlighting the need for better diagnostic and therapeutic options. Finally, in a systematic review, outcome of postnatal systemic corticosteroids (hydrocortisone to dexamethasone) were compared as reported in randomized controlled trials (Boscarino et al.). The authors concluded that dexamethasone appeared to be somewhat more effective than hydrocortisone in improving respiratory outcomes, but with inconclusive but relevant concerns on the uncertainties on long-term neurodevelopmental outcome, again highlighting the need for better therapies for prevention and management of chronic lung disease of prematurity.

### Machine learning or deep learning applications in neonatal care

Artificial intelligence is a rapidly advancing area with fast evolving clinical applications in healthcare, including in the NICU (6). It is no surprise that the current research topic also contains papers illustrating its relevance to improve our practices and outcomes. Two papers hereby focused on NEC, and a 3rd paper on prediction of significant patent ductus arteriosus (PDA). In a mini-review, Cuna et al. reports on the various pathophysiological processes underlying NEC endotypes, and how artificial intelligence holds the promise to influence further understanding and management (Cuna et al.). An approach to enhance surgical decision making in NEC is illustrated by Wu et al. Based on x-rays from 263 neonates diagnosed with NEC (94 surgical cases), a binary diagnostic tool was trained and validated, with Resnet18 as approach applied (Wu et al.). For PDA, an ultrasound-based assessment of ductus arteriosus intimal thickness in the first 24 h after birth was applied in 105 preterm neonates. A prediction model for closure on day 7 included birth weight, mechanical ventilation, left ventricular end-diastolic diameter, and PDA intimal thickness (Hu et al.). Such models can be considered to better target future study, integrated in a precision medicine approach. Use of AI and big data have the potential to significantly improve our understanding of neonatal conditions and also support neonatal researchers in asking better research questions.

### Underreported aspects of contemporary NICU care, holistic care

As part of this research topic, we also accepted papers reporting on the use of music on pain management, on multisensory stimulation to improve maternal milk volume production, and parents' experiences related to congenital cardiac surgery. All these 3 papers reflect the need for holistic care and to further integrate the perspectives of (former) patients and parents into neonatal practice.

In a systematic review, Ou et al. demonstrated that music is an effective intervention to relief procedural pain (e.g., Premature Infant Pain Profile score) in preterm neonates, as it reduced some markers of stress, and improved blood oxygen saturation (Ou et al.). Multisensory stimulation (audiovisual, or audiovisual + olfactory) compared to a control setting improved maternal milk volume production, with evidence of positive effects of both interventions, even more pronounced if both interventions are combined (Cuya et al.). Finally, a quantitative analysis of parent's experiences with neonates admitted to NICU with a congenital heart disease reinformed us on the importance of actively focusing on parental experiences of care (Catapano et al.).

## From advances in neonatal care to implementation

In our opinion, this research topic nicely illustrates the diversity in ongoing clinical research activities, that all hold the promise to improve our clinical management practices, with the overarching aim to improve neonatal outcomes. There is an urgent need to focus on the current health inequities in the provision of care to neonates (3). The trend towards a “neuro” dedicated NICU care is an illustration on how relevant progress may occur. This progress is based on improved neuromonitoring techniques (7), improved management and precision medicine in the field of anti-epileptic drugs (8), and integrating families as partners in neonatal neuro-critical care and similar improvement programs (9). The good news is that we are already experiencing a shift in the right direction. The neonatal community and all other relevant stakeholders need to work better together to improve the pace and scale of this improvement.
